# Physical fitness and nutritional anthropometric status of children from disadvantaged communities in the Nelson Mandela Bay region

**DOI:** 10.17159/2078-516X/2020/v32i1a8158

**Published:** 2020-01-01

**Authors:** D Smith, L Adams, R du Randt, J Degen, S Gall, N Joubert, I Müller, S Nqweniso, U Pühse, P Steinmann, J Utzinger, C Walter, M Gerber

**Affiliations:** 1 Department of Human Movement Science, Nelson Mandela University, Port Elizabeth, South Africa; 2 Department of Sport, Exercise and Health, University of Basel, Basel, Switzerland; 3 Swiss Tropical and Public Health Institute, Basel, Switzerland; 4 University of Basel, Basel, Switzerland

**Keywords:** South Africa, primary schoolchildren, physical fitness, lower socio-economic status, anthropometry

## Abstract

**Background:**

Information about the relationships between physical fitness, body composition and nutrition has increased in recent years; however, little is known about physical fitness and the coexistence of under-/overnutrition among children living in disadvantaged areas.

**Objectives:**

To determine the physical fitness status and its association with body composition, growth and selected socio-demographics in primary schoolchildren from disadvantaged communities in the Nelson Mandela Bay region.

**Methods:**

Nine hundred and sixty-five children (49% girls, M=9.5 years) participated in this cross-sectional study. Height and weight were measured to establish body mass index, and height-for-age z-scores. Physical fitness was assessed using tests from the Eurofit Physical Fitness test battery (flexibility, upper/lower body muscular strength and cardiorespiratory fitness). Between-group differences and cross-sectional associations were examined with univariate (Chi^2^-tests, analyses of variance) and multivariate methods (mixed linear/logistic regression).

**Results:**

Most children had normal weight (76.7%), while 4.5% were underweight and 18.7% were overweight/obese. Underweight children and children with stunted growth (11.5%) had lower average upper body strength (p<0.001). Overweight/obese children had lower scores in weight-bearing activities (p<0.001). Children with higher socio-economic status were more likely to be overweight and obese (p<0.001). In the multivariate analyses, sex, age, body mass index, and stunting were associated with children’s physical fitness.

**Conclusion:**

Fitness assessments seem to be a relevant measure of the current health status of children in disadvantaged settings. Compared to international norms, the children in this study had relatively low scores for both upper- and lower body muscular strength. Therefore, effective school-based intervention programmes should be developed to improve children’s physical fitness in disadvantaged schools.

Globally, an estimated 21% of children under five years old were affected by stunting, while 5.6% of children under five years old were overweight in 2019.^[1]^ In South Africa, a noticeable shift from undernutrition to overnutrition is evident with the rapid economic development and the apparent nutrition transition.^[2]^ Furthermore, it seems that geographic area may be a potential confounder of malnutrition, as different areas may be at different stages of the nutrition transition.^[3]^ Findings from the 2012 South African National Health and Nutrition Examination Survey (SANHANES-1)^[2]^ show that the prevalence of overweight and obesity is highest in urban formal and informal areas, while rural informal locality had the highest prevalence of undernutrition. The literature further highlights an inverse association between stunting and socio-economic status (SES), according to the study by Meko et al.^[3]^ who found stunting to be associated with low SES groups. Malnutrition is of particular concern in low-resourced areas where school tuckshops and vendors outside the school premises offer low-cost food items with little nutritional value.^[2]^ Both forms of malnutrition have adverse health effects and the implications are both immediate and long-term. Children affected by undernutrition are often at higher risk of infectious communicable diseases and are susceptible to physical and cognitive damage that may affect both school and work performance.^[1]^ Overweight and obese children are at an increased risk of non-communicable diseases (NCDs), such as type 2 diabetes, later in life.^[1]^ Malina et al.^[4]^ noted that for children, the functional consequences of malnutrition extend to daily activities, which require movement proficiency. Undernourished children have reduced body size and muscle mass resulting in poorer performance in activities that require muscular strength.^[4]^ Meanwhile, overnutrition reduces aerobic capacity and decreases performance in weight-bearing activities.^[5]^ A systematic review by Ortega et al.^[6]^ provides compelling evidence about cardiorespiratory fitness (CRF) and muscular strength as both physical fitness (PF) components may contribute to the improvement of cardiovascular health in young individuals. Furthermore, PF is a powerful marker of health in childhood and adolescence and may be a predictor for adult morbidity and mortality.^[6]^ However, as far as can be ascertained, no studies have investigated the PF status of primary schoolchildren from disadvantaged communities in the Nelson Mandela Bay region in South Africa.

The main purpose of this paper was to assess the PF and nutritional anthropometric status of Grade 4 schoolchildren from disadvantaged communities in the Nelson Mandela Bay region. Moreover, this study’s goal was to determine the association of PF with selected demographics, such as age, sex, and SES. In previous research, PF has been associated with age, gender and SES. ^[7–8]^ Therefore, this study aimed to determine the association of PF with these selected demographics within this studied population.

## Methods

### Study design

Data presented here were collected in the framework of the Disease, Activity and Schoolchildren’s Health (DASH) study.^[9]^ For the purpose of this paper, a cross-sectional analysis was conducted of the baseline data collected between February and March 2015.

### Participants

The project information was delivered to 103 quintile three government (public) primary schools situated in historically disadvantaged areas of the Nelson Mandela Bay region of South Africa. In South Africa, public schools are classified into five quintiles. Quintile one is the poorest, while quintile five is the least well-off group.^[10]^ The quintile system is linked to the allocation of funds which is determined by the poverty of the community surrounding the school. Eight schools (and 26 classes) were selected, based on the DASH study criteria (at least 100 learners in Grade 4, geographical location [equal number of schools from Northern Areas and Townships], population demographics [similar number of Xhosa-, Afrikaans- and English-speaking schoolchildren), and their willingness to participate. Consent forms were distributed to 1154 children: 145 parents did not consent, resulting in 1009 study participants. Children were informed about the study and provided verbal assent. In total, 965 children met the further inclusion criteria and were considered for the present data analyses. As shown in Table 1, the number of missing values varied across the different indicators. In total, 838 children had complete data on all variables that were used in the present paper.

### Ethical considerations

The DASH study obtained ethical approval from the ethics committee of Northwest and Central Switzerland (EKNZ; 2014–179), the Nelson Mandela University Research Ethics Committee (Human) (H14-HEA-HMS-002), the Eastern Cape Department of Health and the Eastern Cape Department of Education.

### Measures

The components measured to address the primary focus of this paper are PF, socio-demographic background, and anthropometry. PF was assessed using the Eurofit Physical Fitness test battery. Body weight was measured once to the nearest 0.1 kg (Micro T7E electronic platform scale, Optima Electronics; George, South Africa). Height was assessed to the nearest 0.1 cm (Surgical SA; Johannesburg, South Africa). Body weight and height values were used to calculate body mass index (BMI), in which age- and sex- specific cut-offs were applied to determine the prevalence of underweight and overweight/obesity.^[11–12]^ Height values were used to determine height-for-age z-scores (HAZ) according to the World Health Organization’s (WHO) growth standards, and sex-adjusted HAZ scores were used as an indicator for stunting.^[13]^ CRF was assessed using the 20 m shuttle run test, adhering to the protocol by Léger.^[14]^ The number of fully completed laps was recorded when the learner failed to reach the 20 m turn-line on two consecutive intervals. The number of laps was used to calculate the estimated VO_2_max (adjusted for age and sex). Upper body muscular strength was measured by the grip strength test using the Saehan hydraulic hand dynamometer (MSD Europe BVBA; Tisselt, Belgium). Three alternate trials were recorded using a hand dynamometer and averaged. Lower body muscular strength was assessed with the standing broad jump test. The longest of two trial jumps (to the nearest 1 cm) was recorded as the final score. The sit and reach test was used to measure flexibility. The better of two trials (to the nearest 0.1 cm) was recorded as the final score. A detailed description of the procedures can be found in the DASH study protocol.^[9]^ To estimate SES, learners completed a 9-item questionnaire pertaining to durable household asset ownership (e.g. refrigerator) and housing characteristics (e.g. number of bedrooms).^[15]^ A score was calculated based on the dichotomised items (0=not available; 1=available).^[16]^ The items were coded and summarised to build an overall index ranging between 0 and 9. The scores (0–7) represent the lower third of all SES scores and scores (8–9) represents the upper two-thirds of the sample. A principal component analysis with varimax rotation showed that all assessed items loaded reasonably well on the underlying factor (all factor loadings higher than 0.33, 43.3% of explained variance). Moreover, the internal consistency of the SES index was good (Cronbach’s alpha = 0.82).

### Statistical analyses

Quality control ensured the confidentiality, accuracy and completeness of data. The collected data were double-entered and validated in EpiData version 3.1 (EpiData Association; Odense, Denmark). Descriptive statistics and associations were calculated using SPSS version 26 (IBM; Armonk, USA). To examine differences with regard to age, sex, and SES, a series of univariate analyses of variance (ANOVAs) were carried out, with social and demographic background variables as fixed factors. PF and anthropometric measures as dependent variables. χ^2^-tests were performed to examine whether younger vs. older children, boys vs. girls and children with higher vs. lower SES were over-/underrepresented among children who were classified as underweight, overweight/obese or as being stunted. To examine whether children classified as underweight, normal weight or overweight/obese differ from each other with regard to PF, the authors carried out a further series of ANOVAs. Since most children were normal weight and not stunted (see below), Welch tests were used to account for unequal group sizes. Finally, the same procedures were used to examine differences between children classified as stunted vs. not stunted. Bonferroni post-hoc tests were used if more than two groups were compared. To interpret effect sizes, Cohen’s^[17]^ recommendations were followed: η^2^ ≥ 0.01 (small effect), η^2^ ≥ 0.059 (moderate effect), η^2^ ≥ 0.138 (large effect). Finally, to take into account the nested and multivariate nature of the data (students assessed in classes, which are part of schools; interrelatedness between assessed independent variables), mixed linear and mixed logistic regression analyses were performed with random intercepts for school classes. More specifically, sex, age, SES, BMI, and stunting were used to determine the multivariate association with the PF indicators (linear models). Moreover, sex, age, SES, and the PF indicators were used to explain the children’s nutritional status and stunting (logistic models). For mixed linear/logistic regression analyses, the unstandardised B coefficients are provided in combination with the 95% confidence intervals (CI). Across all analyses, the statistical significance was set at p<0.05.

## Results

Descriptive statistics for the total sample are presented in Table 1. The mean age of the children was 9.5 years (SD=1.0), and the sex distribution was similar (48.4% girls, 51.6% boys). Moreover, the study sample was relatively homogeneous with regard to SES. Table 1 also shows that the majority of the children had normal weight (76.8%), whereas 4.5% were underweight and 18.7% were categorised as overweight/obese. Moreover, approximately one in nine children (11.5%) was stunted. Table 1 also summarised the mean scores for all fitness indicators, whereas Table 2 depicts the 50^th^ percentile (P_50_) scores and the interquartile ranges per sex and age for all fitness indicators.

Table 3 summarises the results of the univariate statistical analyses. Younger children were more flexible and had higher VO_2_max values, whereas older children performed significantly better in upper- and lower muscular strength tests. A χ^2^-test showed that children with underweight, normal weight and overweight/obesity were similarly distributed among younger and older children. By contrast, older children (19.2%) had a higher risk of being stunted than younger children (5.7%), χ^2^(1,958)=642.1, p<0.001. Sex-specific differences became apparent with boys outperforming girls in the VO_2_max and muscular strength tests. Girls presented with significantly higher flexibility and BMI values. A Chi^2^-test, χ^2^(2,958)=6.1, p<0.05 indicated that, compared to boys (15.8%), girls were more likely to be classified as overweight/obese (22.0%). By contrast, no significant sex differences were found for stunting. In terms of SES, children with higher SES had significantly higher upper-body muscle strength and significantly higher BMI values. In line with this, children with higher SES scores were overrepresented among overweight/obese children (22.4%) compared to their peers with lower SES scores (11.4%), χ^2^(2,948)=26.6, p<0.001. By contrast, children with lower SES scores had a higher risk (19.0%) of being stunted, compared to their peers with higher SES scores (7.9%), χ^2^(1,948)=25.5, p<0.001.

Table 4 shows that children with varying anthropometric nutritional status differed significantly in the muscular strength and CRF tests. The highest grip strength was observed in children classified as overweight/obese, whereas underweight children had the lowest grip strength. By contrast, overweight children performed significantly worse than their underweight and normal weight peers in the lower body strength and CRF tests. Children classified as stunted had significantly lower upper body muscular strength than their non-stunted peers. None of the other fitness indicators differed between stunted and non-stunted children.

The findings of the multivariate analyses are summarised in Tables 5 and 6. Table 5 shows that girls, younger age and being stunted were associated with better flexibility. Higher upper body muscular strength was associated with boys, older age, higher BMI and being not stunted. Higher scores on lower body muscular strength was associated with boys, older age, lower BMI and being not stunted. Finally, higher CRF was linked to boys, younger age, and lower BMI. In the multivariate analyses, SES was not associated with any of the fitness indicators.

Table 6 shows that older children were more likely to be classified as stunted in the multivariate analyses. Furthermore, after controlling for all other variables, low scores for upper body muscular strength were significantly associated with stunting, whereas no significant association was found with lower body muscular strength and CRF. Table 6 further shows that older peers and children with low upper body muscular strength were more likely to be classified as underweight than normal weight. Moreover, younger children and children with higher upper body and low lower body muscular strength were more likely to be classified as overweight/obese than normal weight. The same also applied for children with lower VO_2_max scores.

## Discussion

In this study, the authors identified the fitness status of primary schoolchildren, examined the association of PF with selected demographics, and investigated the relationship between PF and anthropometric nutritional status. The key findings are that PF indices are associated with children’s social and demographic background. However, children’s sex and age were more closely associated with their PF than their socio-economic background. Moreover, children with lower upper body muscular strength are more likely to be classified as stunted or underweight, whereas children with high upper body muscular strength, low lower body muscular strength, and lower CRF scores are more likely to be classified as overweight/obese.

The results of the multivariate analyses show that higher flexibility was associated with stunting; however, these findings differ from those in Armstrong et al.^[5]^ who reported similar sit-and-reach values regardless of nutritional status. Using the WHO classification for stunting (z<-2),^[13]^ children with stunted growth had significantly lower grip strength; the difference was of moderate magnitude (4.2% of explained variance). This may be due to reduced muscle mass, body size and a deficiency in muscle tissue needed to generate force as a result of early undernutrition.^[4]^ Similar observations were reported in a South African study covering five provinces (Western Cape, Eastern Cape, Gauteng, KwaZulu-Natal and the Free State) where stunted children performed worse than normal weight children in four out of five fitness tests.^[5]^

The findings of the multivariate analyses also showed that overweight/obese children presented with significantly higher grip strength values than underweight and normal weight children. This corresponds with the results found in Malina et al.^[4]^ who reported higher grip strength scores in overweight/obese children, but when grip strength was expressed per unit body mass, then strength was significantly lower in overweight/obese children in comparison to normal weight and stunted children. This confirms that weight has a strong association with grip strength among children. It may be further deduced that the increased grip strength of children with higher SES may be the result of their higher body mass, and thus also higher (absolute, but not relative) muscle mass, since total strength is associated with body mass.^[18]^ This is reflected in the fact that SES was no longer associated with grip strength in the multivariate analyses. Moreover, the univariate analyses in this study identified a nutritional deficiency with SES as children with low SES were at a higher risk of stunting.^[3]^ This is in line with results reported by Meko et al.^[3]^ who confirmed that determining factors for stunting are mainly household food insecurity, low parent education and low employment levels. It is, however, noteworthy that the influence of SES was not apparent in the multivariate analyses, after controlling for other socio-demographic factors and PF indices.

As expected, overweight/obese children performed significantly worse than their underweight and normal weight peers in weight-bearing activities such as the 20 m shuttle run test. This aligns with previous studies which confirm the relationship between the standing broad jump, the 20 m shuttle run and BMI: a higher proportional body mass is associated with lower performance.^[18]^ This study’s results show significant differences in lower body strength and CRF between boys and girls, which may be explained by physiological and anatomical differences. Finally, if compared to international norms derived from tests with European^[19]^ and Australian children,^[20]^ children of the present sample had lower scores for grip strength and standing broad jump, while flexibility and VO_2_max scores were seemingly higher. The low scores in the grip strength and broad jump tests are important because muscular strength proved to be an important health indicator in previous research among children, adolescents and adults.^[6,21]^ For instance, the systematic review by Volaklis et al.^[21]^ reports on the protective role of muscular strength as a modifiable risk factor, as the literature highlights an inverse relationship between muscular strength with all-cause mortality. Therefore, reduced muscular strength performance not only affects functional movement proficiency,^[4]^ but poor muscle strength associated with undernutrition may also increase the risk of metabolic diseases such as lipid disorders and type 2 diabetes.^[21]^ The fact that age and sex were more closely associated with children’s PF than SES may be attributable to the fact that this study’s sample was relatively homogenous in terms of the latter variable.

The strengths of this study are the large sample size, the focus on children from disadvantaged schools, and the large battery of standardised and internationally acknowledged tests. The analyses in this study also went beyond testing univariate relationships and accounted for potential confounders and the nested nature of the data. It can also be assumed that multicollinearity was not an issue in the present analyses, as the highest bivariate correlations between dependent variables was only r=0.42, p<0.001 (between VO_2_max and lower body muscular strength). Moreover, all variance inflation factor (VIF) scores were low (<1.53). Nevertheless, the authors did experience some shortcomings during field testing. For instance, as a self-report measure completed by the children themselves, the SES estimation did have an element of subjectivity. An alternative would have been to assess SES via a parent survey or by using more traditional indicators (such as household income or parental occupation).^[22]^ However, it should be noted that ownership of durable assets, as well as characteristics of housing infrastructure and conditions have been used previously to correctly assess family SES.^[15]^ Importantly, previous studies also showed that children’s self-reports can be used to assess wealth, and that such instruments are able to detect health inequalities across a wide range of different indicators.^[23]^ Furthermore, it is also important to make reference to the testing conditions (e.g. weather, clothing and footwear worn by the children and ground surfaces at the schools), all of which are considered to be limitations that this study had restricted control over. Finally, the focus of this study was on children attending disadvantaged schools. Thus, only limited generalisation is possible for children from more advantaged school settings.

## Conclusion

Fitness assessments seem to be a relevant measure of the current health status of children in disadvantaged settings. Compared to international norms, the children from disadvantaged schools of this study had relatively low scores for both upper- and lower body muscular strength. Therefore, further research and effective school-based intervention programmes should be developed to improve children’s PF in disadvantaged schools.

## Figures and Tables

**Table 1:** Descriptive statistics of all study variables, for the total sample (*N*=965) of quintile three primary schoolchildren in the Nelson Mandela Bay region

Social and demographic background	n	%			
Sex					
Girls	467	48.4			
Boys	498	51.6			
	
	**n**	**Mean**	**SD**	**Min**	**Max**
	
Age (years)	965	9.5	1.0	8.0	15.0
Socio-economic status (0–9)	957	7.5	2.1	0.0	9.0

**Anthropometrics**	**n**	**Mean**	**SD**	**Min**	**Max**

Height (cm)	960	133.2	7.1	109.2	165.3
Weight (kg)	960	30.5	7.6	15.8	87.4
BMI (kg/m^2^)	960	17.0	3.0	11.5	41.7
	
BMI-for-age	**n**	**%**			
	
Underweight	43	4.5			
Normal weight	737	76.8			
Overweight	125	13.0			
Obese	55	5.7			

**Anthropometric status**	**n**	**Mean**	**SD**	**Min**	**Max**

HAZ	960	−0.8	1.1	−4.7	3.1
	
Stunting	**n**	**%**			
	
Not stunted	850	88.5			
Stunted	110	11.5			

**Physical fitness**	**n**	**Mean**	**SD**	**Min**	**Max**

Flexibility (cm)	895	31.3	5.9	9.1	47.6
Upper body muscular strength (kg)	895	12.1	3.1	2.8	25.2
Lower body muscular strength (cm)	891	123.8	19.7	39.0	181.0
CRF (VO_2_max, mL^−1^kg^−1^min^−1^)	874	49.1	4.3	32.3	61.9

The different number of children per variable are due to different number of missing values for different variables. BMI, body mass index; HAZ, height for age; CRF, cardiorespiratory fitness.

**Table 2:** Medians and 25^th^/75^th^ percentiles (quartiles) of grip strength, standing broad jump, flexibility and VO_2_max for quintile three primary schoolchildren in the Nelson Mandela Bay region

			Grip strength (kg)		Standing broad jump (cm)		Flexibility (cm)		CRF (VO_2_max, mL^−1^kg^−1^min^−1^)	

Sex	Age	n	Median	Quartiles	Median	Quartiles	Median	Quartiles	Median	Quartiles
Boys	8	38	11.3	10.1; 13.0	130.0	122.0; 136.0	33.3	30.0; 35.6	51.9	49.7; 56.4
	9	188	12.0	10.2; 13.8	129.0	116.0; 139.0	29.9	29.9; 34.3	50.3	48.0; 54.9
	10	161	13.2	10.8; 14.8	133.5	119.8; 147.0	29.3	25.3; 33.3	51.1	46.3; 53.5
	11	78	14.4	11.8; 16.2	134.0	123.5; 149.0	28.8	25.2; 33.6	52.0	47.0; 54.4
	≥12	19	13.5	10.7; 15.2	135.0	119.5; 148.0	27.6	24.0; 31.9	50.5	46.4; 53.7

Girls	8	66	10.5	9.2; 12.4	114.5	101.3; 125.0	34.9	30.0; 38.4	47.5	47.5; 49.7
	9	244	10.7	10.7; 12.3	117.0	106.0; 128.0	33.1	29.4; 33.1	48.0	45.7; 48.0
	10	113	11.7	9.8; 14.3	122.0	109.0; 133.5	33.8	29.4; 37.1	46.3	43.9; 48.7
	11	35	12.9	10.9; 14.7	121.0	101.8; 132.5	31.5	27.7; 35.6	44.6	42.1; 47.0
	≥12	9	14.3	12.1; 15.3	120.0	110.5; 127.0	31.6	24.4; 34.3	42.9	38.3; 47.9

**Table 3:** Comparison of physical fitness parameters, based on children’s age, sex and socio-economic status amongst the study population from quintile three primary schools in the Nelson Mandela Bay region

	Age						

	8–9 years (n=550)		10–15 years (n=415)				
				
	Mean	SD	Mean	SD	F	p	η^2^
	
Flexibility (cm)	31.9	5.7	30.3	6.2	16.3	<0.001	0.018
Upper body muscular strength (kg)	11.3	2.8	13.1	3.2	79.9	<0.001	0.082
Lower body muscular strength (cm)	120.6	19.0	128.2	19.9	33.4	<0.001	0.036
CRF (VO_2_ max, mL^−1^kg^−1^min^−1^)	49.4	3.9	48.8	4.8	4.2	0.042	0.005
BMI (kg/m^2^)	17.0	3.0	17.0	3.0	0.0	0.994	0.000

	**Sex**						

	**Boys (n=498)**		**Girls (n=467)**				
				
	**Mean**	**SD**	**Mean**	**SD**	**F**	**p**	η^2^
	
Flexibility (cm)	29.7	5.8	32.9	5.6	73.5	<0.001	0.076
Upper body muscular strength (kg)	12.8	3.1	11.3	2.9	52.1	<0.001	0.055
Lower body muscular strength (cm)	130.6	19.1	116.8	17.8	123.3	<0.001	0.122
CRF (VO_2_ max, mL^−1^kg^−1^min^−1^)	50.9	4.4	47.4	3.4	174.6	<0.001	0.167
BMI (kg/m^2^)	16.8	2.7	17.2	3.3	4.0	0.046	0.004

	**Socio-economic status (SES)**						

	**Lower SES (0–7) (n=317)**		**Higher SES (8–9) (n=640)**				
				
	**Mean**	**SD**	**Mean**	**SD**	**F**	**p**	η^2^
	
							
Flexibility (cm)	31.1	5.8	31.4	6.0	0.5	0.502	0.001
Upper body muscular strength (kg)	11.4	2.9	12.4	3.1	18.4	<0.001	0.020
Lower body muscular strength (cm)	125.0	17.6	123.4	20.7	1.2	0.269	0.001
CRF (VO_2_ max, mL^−1^kg^−1^min^−1^)	49.0	4.4	49.2	4.2	0.5	0.468	0.001
BMI (kg/m2)	16.4	2.7	17.3	3.1	21.2	<0.001	0.022

CRF, cardiorespiratory fitness; BMI, body mass index

**Table 4:** Comparison of fitness parameters among children who are underweight, normal weight and overweight/obese, or stunted versus not stunted, attending quintile three primary schools in the Nelson Mandela Bay region

	Underweight (1)		Normal weight (2)		Overweight/ Obesity (3)		Welch-test			Bonferroni post-hoc comparison		

	Mean	SD	Mean	SD	Mean	SD	F	p	η^2^	1–2	1–3	2–3
	
Flexibility (cm)	30.4	5.9	31.1	6.0	32.2	5.8	2.6	0.075	0.006	1.00	0.283	0.115
Upper-body muscular strength (kg)	9.8	2.6	11.8	2.9	13.5	3.4	29.2	<0.001	0.065	<0.001	<0.001	<0.001
Lower-body muscular strength (cm)	125.5	19.8	126.7	18.6	111.6	19.9	39.3	<0.001	0.089	1.00	<0.001	<0.001
CRF (VO_2_max, mL^−1^kg^−1^min^−1^)	49.1	4.0	49.7	4.3	47.0	3.8	31.6	<0.001	0.060	1.00	0.012	<0.001

	**Not stunted**		**Stunted**				**Welch-test**					

	**Mean**	**SD**	**Mean**	**SD**			**F**	**p**	**η** **^2^**			
	
Flexibility (cm)	31.3	6.0	31.4	5.9			0.0	0.867	0.000			
Upper-body muscular strength (kg)	12.3	3.1	10.3	2.6			48.9	<0.001	0.041			
Lower-body muscular strength (cm)	123.8	20.3	123.7	15.4			0.0	0.929	0.000			
CRF (VO_2_max, mL^−1^kg^−1^min^−1^)	49.2	4.2	49.0	4.9			0.2	0.615	0.000			

CRF, cardiorespiratory fitness

**Table 5:** Association between physical fitness indices as well as sociodemographic background variables and anthropometric nutritional status among children attending quintile three primary schools in the Nelson Mandela Bay region

	Physical fitness indices											

	Flexibility (cm)			Upper body muscular strength (kg)			Lower body muscular strength (cm)			CFR (VO_2_max, mL^−1^kg^−1^min^−1^)		
	
	B	Estimate (95% CI)	p	B	Estimate (95% CI)	p	B	Estimate (95% CI)	p	B	Estimate (95% CI)	**p**
	
**Sex**												
Girls (reference)	—			—			—			—		
Boys	−3.02	−3.76; −2.28	<0.001	1.22	0.88; 1.56	<0.001	11.51	9.14; 13.88	<0.001	3.66	3.17; 4.16	<0.001
**Age (years)**	−0.57	−0.99; −0.14	<0.001	1.19	1.00; 1.38	<0.001	2.98	1.64; 4.31	<0.001	−0.93	−1.21; −0.65	<0.001
**Socio-economic status (0–9)**	−0.09	−0.28; 0.10	0.341	0.08	−0.01; 0.16	0.084	0.27	−0.33; 0.86	0.377	−0.05	−0.18; 0.07	0.429
**BMI (kg/m** **^2^** **)**	0.01	−0.11; 0.14	0.835	0.33	0.27; 0.39	<0.001	−2.02	−2.42; −1.61	<0.001	−0.45	−0.54; −0.37	<0.001
**Stunting**												
Stunted (reference)	—			—			—			—		
Not stunted	−1.44	−2.66; −0.22	0.021	1.92	1.36; 2.47	<0.001	5.61	1.70; 9.51	0.005	0.01	−0.80; 0.82	0.976
**Overall model information**	Corrected model: F(5,873)=17.6, p<0.001 Constant term: B=40.1, Estimate (95% CI): 35.0; 45.2, T=15.6, p<0.001			Corrected model: F(5,873)=81.6, p<0.001 Constant term: B=−7.8, Estimate (95% CI): −10.1, −5.5 T=−6.7, p<0.001			Corrected model: F(5,869)=48.5, p<0.001 Constant term: B=116.9, Estimate (95% CI): 10.9; 132.8, T=14.4, p<0.001			Corrected model: F(5,852)=73.6, p<0.001 Constant term: B=64.2, Estimate (95% CI): 60.8; 67.5, T=37.7, p<0.001		

In the mixed linear regression models, cases were excluded listwise from the analysis if they had missing data in one or several of the covariates or in the dependent variables. Therefore, the analyses were based on the following number of students: flexibility: n=879; upper body muscular strength: n=879; lower body muscular strength: n=875; cardiorespiratory fitness: n=858. B is the adjusted unstandardized estimate of the Beta coefficient. Estimate (95% CI) is the adjusted unstandardized Beta coefficients, 95% confidence interval. All p-values are calculated using mixed linear regression, adjusting for clustering of school classes. CFR, cardiorespiratory fitness; BMI, body mass index

**Table 6:** Associations between anthropometric nutritional status as well as socio-demographic background variables and physical fitness indices among children attending quintile three primary schools in the Nelson Mandela Bay region

	Mixed logistic (binary) regression analysis			Mixed logistic (multinomial) regression analysis					

	Stunted vs. not stunted (reference category)			Underweight vs. normal weight (reference category)			Overweight/obese vs. normal weight (reference category)		
	
	B	Estimate (95% CI)	p	B	Estimate (95% CI)	p	B	Estimate (95% CI)	p
	
**Sex**									
Girls (reference)	—			—			—		
Boys	0.26	−0.35; 0.87	0.405	−0.42	−1.30; 0.47	0.357	0.55	0.04; 1.05	0.034
**Age (years)**	1.37	1.02; 1.72	<0.001	0.74	0.32; 1.15	<0.001	−0.91	−1.22; −0.60	<0.001
**Socio-economic status (0–9)**	−0.05	−0.17; 0.56	0.332	−0.07	−0.22; 0.09	0.414	0.07	−0.06; 0.19	0.286
**BMI**									
Overweight/obese (reference)	—			—			—		
Normal weight	1.55	0.05; 3.04	0.042	—	—	—	—	—	—
Underweight	1.56	−0.17; 3.29	0.078	—	—	—	—	—	—
**Stunting**									
Stunted (reference)	—			—			—		
Not stunted	—	—	—	−0.25	−1.12; 0.62	0.577	1.20	−0.28; 2.68	0.111
**Flexibility (cm)**	0.08	0.03; 0.13	0.002	−0.02	−0.08; 0.05	0.600	0.04	0.00; 0.08	0.035
**Upper-body muscular strength (kg)**	−0.44	−0.56; −0.31	<0.001	−0.37	−0.54; −0.20	<0.001	0.32	0.24; 0.40	<0.001
**Lower-body muscular strength (cm)**	−0.01	−0.03; 0.01	0.297	0.01	−0.01; 0.04	0.295	−0.04	−0.05; −0.03	<0.001
**CRF (VO** **_2_** **max, mL** **^−1^** **kg** **^−1^** **min** **^−1^** **)**	0.05	−0.02; 0.11	0.190	0.03	−0.07; 0.12	0.590	−0.21	−0.29; −0.15	<0.001

**Overall model information**	Corrected model: F(9,820)=9.9, p<0.001 Constant term: B=−15.4, Estimate (95% CI): −20.6; −10.2, T=−5.8, p<0.001			Corrected model: F(16,820)=10.1, p<0.001. Normal vs. overweight: Constant term: B=−7.6, Estimate (95% CI): −14.9; −0.3, T=−2.0, p=0.042. Normal vs. overweight: Constant term: B=14.6, Estimate (95% CI): 9.2; 20.0, T=5.3, p<0.001					

In the mixed logistic regression models, cases were excluded listwise from the analysis if they had missing data in one or several of the covariates or in the dependent variables. Therefore, the analyses were based on the following number of students: stunting: n=838, BMI status: n=838. B is the adjusted unstandardized estimate of the Beta coefficient. Estimate (95% CI) are the unstandardized Beta coefficients, 95% confidence interval. All p-values are calculated using mixed linear regression, adjusting for clustering of school classes. CFR, cardiorespiratory fitness; BMI, body mass index
